# Advancing health equity for Roma people in Romania

**DOI:** 10.1371/journal.pgph.0006692

**Published:** 2026-06-25

**Authors:** Margareta Matache, Eugene Richardson

**Affiliations:** University of Oslo Faculty of Medicine: Universitetet i Oslo Det medisinske fakultet, NORWAY; 1 Harvard T. H. Chan School of Public Health, Department of Social and Behavioral Sciences, Boston, Massachusetts, United States of America; 2 Assistant Professor of Global Health and Social Medicine, Harvard Medical School, Boston, Massachusetts, United States of America; 3 Assistant Professor of Medicine, Brigham and Women’s Hospital, Boston, Massachusetts, United States of America

## Abstract

Over the past few decades, scholars in the humanities and social sciences have increasingly interrogated the histories and legacies of violence against Romani people through critical, decolonial, feminist, and anti-racist lenses. Building on this body of research, this article examines continuities of anti-Roma racism in a specific place, Romania, and how they can help reproduce and shape health outcomes and inequities today. Using a critical analysis of Romanian laws, policies, literature, and public discourse, we propose the right to a remedy for past collective injustices faced by historically oppressed and racialized populations as a pathway to the full realization of the right to health in places marked by continuous collective injustices. We argue that the realization of the right to health must be understood and addressed beyond equal access and protections against violations of the right to health; it also requires addressing the legacies and persistence of anti-Roma racism and its long-term impacts on health, well-being, and quality of life, as well as addressing other enduring oppressions.

## Introduction

Across Europe, from the era of sovereign rule to that of nation-states, Romani people have been subjected to multiple and enduring forms of violence, exclusion, and discrimination [1,2]. Against this backdrop, scholars across the social sciences and humanities have examined both the history of this oppression and its impacts. Moreover, the growing attention to human rights in post-communist Central and Eastern European countries has led to increased documentation of human rights violations, and consequently, to a broader body of knowledge on anti-Roma injustice [3–6]. Furthermore, recent scholarship has framed anti-Roma racism not as a product of ideology and prejudice, but as a form of structural violence [7] and an entrenched technology of power [1,8] that has long structured the distribution of power, resources, and privilege.

Research on the multiple and interlinked impacts of racism on Romani people’s health and well-being, however, remains limited. The available quantitative research, nonetheless, points to differential health outcomes and unequal access to healthcare across European countries with large Romani populations [9,10]. In Romania, for example, some of the most striking health indicators are low life expectancy and high premature mortality rates. Roma live seven years less on average than the general Romanian population [11] and infant mortality is four times the national average [12]. Such differences are not cultural, biological, or genetic; they are produced by social, economic, geographical, environmental, and political conditions, thus avoidable and unjust.

Yet explaining these inequities requires a broader understanding of racism than the one that often prevails in European institutions and scholarship. In Europe, racism is typically limited to ideologies, beliefs, discriminatory acts, or individuals, including those in institutions. And indeed, anti-Roma racism manifests in overt, covert, intentional, unintentional, interpersonal, institutional, violent, passive, and other forms. Racism manifests in acts of discrimination, hate speech, and prejudice. Discrimination is a visible expression of racism. However, understanding anti-Roma racism only through this liberal approach ignores the long history through which Romani people have been excluded from the conceptualization and practice of “European humanness.” Anti-Roma racism persists, adapting and reproducing itself in liberal institutions, neighborhoods, and spaces, often in the absence of overt racist ideologies or stereotypes. In this sense, anti-Roma racism operates predominantly as a structural form of oppression [1].

Global public health scholarship shows that health inequities are connected, among other structural, environmental, social, and political factors, to racism [13–16]. A significant body of research establishes that stressors related to racism generate and contribute to trauma, early death, poor health outcomes and behaviors, and socioeconomic inequities [15,16]. Moreover, unrepaired historical harm, with its enduring legacies of power imbalances and socioeconomic and wealth inequities, continues to disadvantage racialized individuals and families [13,17]. These factors intersect with, and further impact the availability, accessibility, acceptability, and quality of health (AAAQ) – the basic elements of the right to health as established by the Committee on Economic, Social and Cultural Rights.

This article examines both contemporary and historical manifestations of anti-Roma racism that have impeded health and well-being, and thus focuses on the historical and institutional dimensions of racism in the territories of present-day Romania. This specific geographic focus is motivated both by the size of Romania’s Romani population (estimated at 1.8 million and making up at least 8% of the Romanian population [18]) and by the particularly continuous and severe history of anti-Roma racism. Still, Romani families and neighborhoods in Romania, like any other population group, are heterogeneous across subgroups, class, gender, sexuality, religion, and the degree of exposure to state racism. Thus, our argument is not that Romani people experience racism the same way, but that anti-Roma racism structures their exposure to harm.

We argue that the realization of the right to health requires both the protection of rights and access to remedies guaranteed by international human rights norms, as well as collective reparative measures, as proposed by the recent UN Report of the Working Group of Experts on People of African Descent [19], which addresses historical collective injustices.

We suggest that a comprehensive assessment of the realization of the AAAQ standards for health among Romanian Roma, requires: first, recognition and measurement of anti-Roma racism as a continuous, direct, and structural determinant of health; second, recognition of the direct impacts of human rights violations on health outcomes; and third, the broadening of the right to remedy to historical injustices as a pathway to both reparatory justice and health equity.

This analysis draws on the existing literature, adding a critical analysis of policies and public discourse concerning Romani people, especially those related to violations of the right to health, structural oppressions, and the right to remedies for historical injustices. Given the lack of official disaggregated data by ethnicity, we acknowledge limitations in identifying causal links between historical collective injustices, ongoing human rights violations in the AAAQ of health, and health inequities. Still, where relevant, we draw on scholarship from other contexts of historical injustices that helps inquire and suggest such connections.

## Harms shaping life and health in historical perspective: Bodily violence, starvation, and reproductive violence

Anti-Roma racism has persisted, though it has also transformed throughout Romanian history. From the late Middle Ages to the present, Romani individuals and families, particularly those enslaved, exploited, impoverished, and forced into residentially segregated neighborhoods and villages, have faced multiple forms of institutionalized violence, discrimination, and disinvestment, as well as politics of inaction and neglect [1,2,20–22].

Many of its cumulative physical, psychological, sexual, reproductive, cultural, social, and economic harms remain largely unquantified. Yet the absence of disaggregated data and precise measurements should not prevent the acknowledgment of the historical record of recurring harms with evident, direct consequences for health and well-being [1,2,7,20,21,23–28].

This section briefly explores three interconnected types of harm through which anti-Roma racism has influenced health over time: bodily violence, starvation, and reproductive violence. We don’t quantify impacts, but we trace these harms across political and historical sequences - from the feudal order of enslavement through the eugenic era, the Nazi-aligned regime, and state socialism – to show specificities, continuities, and transformations.

### Bodily violence

Bodily violence is one of the more overt ways in which racism impacts health and well-being.

In the historical principalities of Moldavia and Wallachia, historical territories of present-day Romania, Roma were forced into institutionalized enslavement for nearly five hundred years, beginning in the 1300s. The voivodeships, Orthodox monasteries, and the aristocracy - the primary categories of enslavers - gradually established and institutionalized this racialized system of labor exploitation.

During slavery, bodily violence materialized not only through forced, unpaid, and inhumane labor but also through physical and psychological abuse and torture. The Custom of the Land (customary or unwritten laws regarding land ownership, property, distribution, and agriculture), followed by varied written laws, as well as common practices, allowed for physical violence against enslaved individuals. Hence, beatings and torture of enslaved people, including children, were common. Moreover, although the Custom of the Land and the subsequent written laws technically prohibited killings, in practice, murdering enslaved people went mostly unpunished and unaccounted for [1,2,23,29].

Bodily violence was central to slavery, with enslavers using brutal methods like torture, beatings, and abuse against enslaved Roma who worked, breastfed, and cared for families daily. While punishment was an apparent reason, violence also reinforced social and racial hierarchies, deterred escapes, rage, and revolts, and fostered terror to maintain subordination [1].

Bodily violence did not end with the abolition of this system of racialized slavery. During the twentieth century, the Romanian Nazi-aligned regime also enforced bodily harm through a blatant institutionalized project of deportation camps in Transnistria; approximately 25,000 Roma were deported and 11,000 died [30].

While bodily violence was central to the operation of slavery, during the Holocaust, it also became an end in itself. However, the frequency, extent, and impact of bodily violence on Romani individuals’ health and lives during slavery and the Holocaust remain unknown. Yet, present-day theories and data on abuse show, for instance, “a strong graded relationship” between childhood abuse - emotional, physical, or sexual - and early death, health risk behaviors, or disease [31]. Thus, although we cannot measure the full scale of these harms, the historical record and contemporary evidence suggest severe consequences on health and well-being.

### Starvation

Starvation acts as another form of violence through which racism directly affects health.

During racialized slavery in Moldavia and Wallachia, starvation was used both to control wealth and power and as a form of punishment, sometimes leading to death. As Mihail Kogălniceanu, an abolitionist, former enslaver, and historian, documented in the late 1800s, “(…) starvation, being hung over smoking fires, solitary imprisonment and being thrown naked into the snow or the frozen rivers, such was the fate of the wretched G*psy” [32,33].

Institutionalized starvation reemerged with devastating force during the Holocaust. In Transnistria, where Roma were forced into camps, the food rations were sometimes withheld from them for weeks. Roma were also not given firewood, preventing them from preparing food or staying warm [21,30]. The politics of death were enforced by the circumstances of life, rather than through direct extermination.

The frequency, duration, and effects of starvation on health and well-being also remain unknown. However, starvation is an evident life-threatening condition that causes physical harm. Research in other historical and geographic contexts, such as the Dutch Hunger Winter, shows that “the in utero window is most vulnerable to malnutrition effects that lead to physiological consequences consistent with metabolic syndrome in later life (e.g., diabetes, cardiovascular disease, obesity) as well as muscular-skeletal deficiencies and auditory impairment. Conversely, for cohorts exposed at later developmental stages (childhood and adolescence), results suggest a resilience to the effects of malnutrition on physical health in late life, but a higher vulnerability with regard to socioeconomic indicators” [34].

Once more, although the extent of the harm also remains difficult to quantify, historical records and current evidence indicate that the starvation of Roma by different political regimes also led to severe health and well-being consequences.

Starvation served as both a form of punishment and a means of wealth during slavery. However, during the Holocaust, it also became a tool in the politics of death.

## Reproductive violence

Reproductive violence represented a third significant category of harm through which anti-Roma racism has affected health and well-being throughout political regimes.

Under slavery, reproductive violence was inflicted especially through sexual violence and the separation of children from their families. Sexual violence was inflicted by both ordinary people and enslavers, but enslavers had more power and purpose in using it [26,27]. Enslaved girls were raped, sometimes by the sons of enslavers, as a form of sexual “apprenticeship” [35]. Enslavers cast Romani girls and women as “breeding females,” subjecting them to sexual abuse and regulating their marriages for wealth accumulation and political influence [20,26,27]. At the same time, enslavers also separated Romani families from their children. By law, enslavers claimed ‘ownership’ of Romani children at birth as their property, and those who did not have a ‘master’ became the property of the Crown. Enslaved children could be separated from their parents to be sold, gifted, or donated as commodities and an exploitable labor force [2,21,23,28,33].

Reproductive violence did not disappear after slavery either. While sexual violence continued during the Holocaust, with the onset of state-socialism in 1947, other distinct and often veiled forms of reproductive violence emerged. For instance, the communist party put in place a gendered and racialized biopolitical project of reproductive control [36]. While it imposed a strict anti-abortion policy at the national level starting in the 1970s, it had a more flexible, yet racialized approach toward Romani women, rationalized through *mythcrafting* [1]: “[t]heir exaggerated reproduction is determined especially by their lifestyle, the degree of their social and cultural backwardness” [37].

The impacts of reproductive violence across slavery, the Holocaust, and state-socialism on health also remain unknown. Yet, global research shows that sexual violence and child-family separation control, coerce, and punish women and other genders, with serious physical and mental health consequences. Thus, when considering the historical record alongside contemporary evidence, there is another robust indication of severe impacts on health, bodily autonomy, family life, and overall well-being.

Moreover, child-family separation disrupts the mother-child bond. As sociologist Dorothy Roberts argues, it denies mothers the right to nurture, raise, and remain with their children, devalues and inferiorizes them, and undermines their reproductive roles and abilities [38], impacting physical and mental health and well-being.

Taken together, these historical harms suggest that anti-Roma racism in Romania has manifested not only through random individual acts of prejudice and discrimination but more so through recurring and organized politics of bodily harm, starvation, and reproductive violence. The scale and severity of death, premature mortality, disease, and trauma, along with the multiple health, socioeconomic, collective, and personal losses resulting from these historical collective injustices, remain largely overlooked and unmeasured. Still, global research has already linked historical injustices to present-day health and socio-economic inequities and health disparities [17,39,40]. In this sense, racialized slavery [2,23], eugenic politics [41], the Holocaust [30,42], and state-socialism [36] should be understood as historical harms that shaped Romani people’s health, well-being, bodily autonomy, family life, human rights, potential, and opportunities over time.

## The right to health and health equity

Harms to health and well-being are not only a matter of the past. Anti-Roma racism has gradually been embedded in our systems and society, persisting through overt laws and implicit privileges across cultural, social, political, and economic spheres. And today, gadjikane — gadjo-led and normative – politics and policymaking are key to maintaining this anti-Roma oppression in place [1].

Power, human hierarchies, and dehumanization, now normalized and invisible, continue to shape ideologies, policies, and practices. While aiming for individual rights and equal opportunities, liberal democracies often remain unfair to historically marginalized groups. Although societal norms and laws promote equal rights for all, they mainly serve the interests of the in-power groups [1].

In liberal democratic Romania, institutional anti-Roma racism continues to shape Roma health through both old and new manifestations, such as bodily violence, reproductive violence, discriminatory treatment in healthcare institutions, and unjust access to material and territorial conditions necessary for health. Together, these mechanisms indicate that historical injustices intersect with present-day injustices. In what follows, we will briefly examine remnants, continuities, and transformations in these patterns.

### Bodily violence

Bodily violence remains one of the most overt ways in which anti-Roma racism manifests in liberal democracy in Romania. In particular, violence perpetrated by state actors, particularly the police, has been a recurring issue. Between 2006 and 2015, the Roma Center for Social Interventions and Studies - Romani CRISS documented 48 police brutality cases in Romania - seven Roma killed, 187 beaten or tortured. Some of these abuses included children [3]. While Romanian courts rejected all complaints from victims, the European Court of Human Rights recognized the harms and condemned the state for failing to deliver justice in cases of police abuse it reviewed.

### Reproductive violence

Reproductive violence remains a concern, although its present-day forms in Romania differ from those documented in the past and those in other parts of contemporary Central and Eastern Europe. In contrast with the Czech Republic and Slovakia [4,43]. Forced sterilization does not seem to be a common institutionalized practice in Romania, although it may sporadically occur. Such an alleged case was discussed in 2019 by the New York Times, which reported on the separation of a four-month-old Romanian Roma baby from his parents. Emphasizing the mother’s experience in Romania, the article noted that “in a haze of pain while she was in labor, Florentina signed documents that she couldn’t read. When she returned to the hospital for an appointment to check on her recovery, a hospital employee told her that the doctor had also performed a tubal ligation. She and her husband had planned to have more children, as is traditional in their culture. They were devastated” [44].

Discursive support for reproductive control is occasionally visible in public life as well. In 2013, an extremist organization in Timisoara publicly offered the equivalent of approximately $90 “to each G*psy woman in the Banat area” who agreed to be sterilized [45], In 2020, a municipal mayor recommended screening the educational and income level of Roma parents before allowing them to have children [46].

In turn, child-family separation continues to be a pressing issue of reproductive violence in Romania. Romani children are disproportionately removed from their parents compared to non-Romani children. While recent research does not exist, in 2011, the European Roma Rights Center reported that “28% of children in institutional care are Roma, even though they make up just 9% of the population.” They also underlined that child protection workers and social workers treat poverty as a parental failure rather than systemic exclusion [5,28].

### Discrimination within healthcare settings

Institutional discrimination within healthcare settings is an additional mechanism through which anti-Roma racism undermines the right to health.

Global public health research shows that everyday and major discrimination poses major threats to individuals’ health and well-being [15,16]. Yet, in Romania’s neoliberal democracy, overt and covert discriminatory practices occur in the very institutions that ought to ensure adequate and equal health, medical, and hospital care and services.

One apparent pattern of discrimination in health institutions is segregation in hospital wards. In 2014, Roma NGOs provided the UN with evidence documenting the practice of segregating Roma patients in hospitals: the Roma women’s wards were “unrenovated...and untidy,” nurses would very rarely change the sheets, and babies were given medicines, using unsterilized utensils that had already been used for other patients [6]. Similarly, Romani children were segregated in special wards in a major Bucharest children’s hospital [47]. These examples are not national policies but rather institutionalized practices reinforced through local discretion and limited accountability.

Romani patients also experience discrimination in emergency departments, with hospital staff following distinct processes of orienting, healing, and interacting with them. Research suggests that hospital staff often follow different practices and processes when orienting, treating, and interacting with Romani patients, leading to longer wait times for Romani patients compared to non-Romani individuals, among other issues. Biases, whether conscious or unconscious, appear to inform differential behaviors: “the staff’s general perception is that Romani patients’ aggression, ranging from physical violence to interactional insubordination and defiant conduct, is pervasive and routine” [48].

Romani patients themselves experience and recognize these behaviors as discriminatory. In a 2017 survey, 68 percent of Romanian Roma respondents reported experiencing negative attitudes from healthcare providers [12]. A 2023 study shows that 16% of the interviewed Romanian Roma felt discriminated against when accessing health services [9].

### Unequal access to care and insurance

Unequal access to healthcare and health-related resources constitutes another barrier. More than half of Romania’s Romani population lacks access to a medical facility/pharmacy. Moreover, 58 percent of Roma have access to health insurance [9]. According to a 2012 UNDP study, only 51 percent of adult Roma (16+) had health insurance, compared to 85 percent of the majority population, and 71 percent of Roma could not afford medicine compared to 31 percent of non-Roma [10]. These disparities indicate that the problem is not discrimination only, but also unequal access to basic services and care.

These imbalances in access are tightly linked to old and new socioeconomic, environmental, political, and structural barriers. Critically, almost 80 percent of Roma are at risk of poverty compared to 35 percent of non-Roma [49]. Nutrition is inferior to that of the in-power group [50]. Two-thirds of Roma (compared with 38 percent of the majority) live in households without running water, and an even higher percentage have no toilet or bathroom in the house [51]. More than half of Romanian Roma live in poorly resourced and residentially segregated neighborhoods, some living “in poor houses or slums” [52], a reality which can be partly attributed to a legacy of slavery in the regions where slavery existed. Yet, as the United Nations Special Rapporteur on extreme poverty and human rights reported in 2015, “many Romanian officials are in a state of denial about the extent of poverty and discrimination against the extremely poor, especially the Roma” [53].

### Residential segregation

Romanian research and official data have neglected to develop metrics that measure residential segregation, among other factors, as a determinant of health outcomes and inequities. Nonetheless, eco-social theorists show that adverse social, environmental, economic, and structural conditions directly impact individuals’ health [54]. In this sense, the lack of or limited healthcare resources and facilities in residentially segregated Romani neighborhoods, which face systemic disinvestment, can also function as a driver of health inequities and adverse health outcomes.

Thus, anti-Roma racism in contemporary Romania operates through both episodic human rights violations and institutional routines, systematically undermining healthcare availability, accessibility, acceptability, and quality for Roma. In this context, health inequities transpire across many measurable indicators, with Roma faring far worse in recorded major health outcomes and access to health services than the rest of the Romanian population [9,37].

## Moving Forward. Broadening the right to remedy

When these records of injustice are read alongside contemporary health evidence, we can suggest that health inequities among Roma in Romania are perpetuated not just by isolated rights violations but also through a combination of historical collective injustices, institutional discrimination, residential segregation, and material deprivation. This suggests that any adequate remedy should be conceived at the individual, family, institutional, and chrono-structural levels.

Contemporary human rights frameworks guarantee the right to justice and remedy for individual human rights violations, as well as gross violations of human rights and serious breaches of international humanitarian law. Romani individuals and civil society have brought cases of human rights violations before national and European courts, with some victims receiving justice.

In other countries, the right to remedy at both individual and collective levels has been addressed in the case of forced sterilization of Romani women during communist Czechoslovakia and, later, in liberal Czech Republic and Slovakia. In the case of Slovakia, the European Court of Human Rights found Slovakia in violation of the right to autonomy and choice, as well as freedom from inhumane and degrading treatment, for eight women who filed complaints and ordered compensation. In 2021, the government also issued a formal apology. However, the former human rights commissioner for the Council of Europe welcomed the symbolic steps but called for reparations for victims of forced or coercive sterilization [55]. Similarly, in the Czech Republic, the government apologized in 2009, and in 2012, the Parliament adopted a compensation bill for women unlawfully sterilized from 1966 to 2012 [4,47].

These developments nevertheless reveal important limitations in the implementation of the right to remedy, including insufficient or no attention to the accountability of perpetrators and the empowerment and vindication of all victims. More broadly, they expose the limits of the liberal framework centered on individual human rights in addressing collective injustices.

Romania has not implemented similar remedy mechanisms, but these precedents are relevant because they clarify the kinds of reparative measures that such violations may warrant. Moreover, these precedents are relevant because any comparable remedies in the Romanian context would also need to account for police brutality, family separation, hospital segregation, discriminatory emergency care, and unequal access to healthcare services.

The right to remedy for the consequences of historical collective injustices has long been debated, but it has not yet been guaranteed by global or regional international legal frameworks. In the case of historical injustices targeting Romani people, the methods of backward-looking reparations [56] can most plausibly be determined through the involvement of victims’ descendants in participatory decision-making processes. Moreover, some conventional methods of reparations utilized in other contexts [56–61] may also be of use in ensuring the right to remedy, including monetary compensation, restitution, or apologies [11]. However, evidence from other contexts suggests that the right to remedy for historical collective injustices should extend beyond backward-looking approaches and symbolic and educational acts.

Research and advocacy on reparations point to one important component of a broader, forward-looking remedial framework: research and community-led consultations on how the history of slavery, eugenics, and the Holocaust, and the continuities of reproductive control, residential segregation, and institutional discrimination, shape current health inequities. Critically, such research findings, along with community consultations, could provide the basis for reparatory measures, particularly in health.

One implication would be the effective allocation of financial resources to ensure that Romani individuals, particularly those living in residentially segregated neighborhoods and villages, benefit from the availability, accessibility, acceptability, and quality of health in any clinics, hospitals, and pharmacies. Such measures may include the assessment of the AAAQ elements of the right to health and the demand/need for free mental and physical health services, as well as the establishment of community centers and support groups.

While the full realization of the right to health depends on both protecting human rights and establishing a right to remedy for historical injustices, these alone cannot address all the barriers related to the AAAQ of healthcare for Roma individuals, families, and neighborhoods. Ultimately, advancing health equity in Romania requires more than protection against human rights violations; it requires a framework that expands to include reparative justice: one that recognizes, measures, and tackles human rights violations and identifies, measures, and addresses structural inequities, including through anti-racist interventions, health data disaggregation, Roma leadership, and fair redistribution of power, resources, and prestige.
